# New SNAP Eligibility in California Associated With Improved Food Security and Health

**DOI:** 10.5888/pcd18.200587

**Published:** 2021-04-01

**Authors:** Melinda Wang, Ronli Levi, Hilary Seligman

**Affiliations:** 1 Yale University School of Medicine, New Haven, Connecticut; 2 University of California San Francisco, Division of General Internal Medicine, Department of Medicine, San Francisco, California; 3 Center for Vulnerable Populations at Zuckerberg San Francisco General Hospital, San Francisco, California

## Abstract

**Introduction:**

In California, Supplemental Security Income beneficiaries were ineligible to receive Supplemental Nutrition Assistance Program (SNAP) benefits until a June 2019 policy change. The objective of this study was to determine whether SNAP eligibility was associated with changes in food insecurity and health among older adults and adults with disabilities.

**Methods:**

We administered a survey to SSI recipients (N = 213) before (May–August 2019) and after (September 2019–January 2020) the policy change. We examined changes in food insecurity (primary outcome), health status, stress, medication adherence, and dietary intake from baseline to follow-up. Multivariable analyses adjusted for age, sex/gender, race/ethnicity, and education.

**Results:**

Of 213 participants at baseline, 56.8% were male, 43.7% were Black/African American, 88.7% had an annual income of less than $15,000, and 89.7% were currently housed. Of 157 participants at follow-up, 114 (72.6%) were newly enrolled in SNAP. At follow-up, compared with baseline, participants were less likely to report food insecurity (83.1% vs 67.5%, *P* < .001), required less additional money for food ($73.33 vs $47.72 weekly, *P* < .001), were more likely to report excellent/very good health (26.8% vs 27.6%, *P* < .001), and were less likely to report cost-related medication nonadherence (24.1% vs 17.7%, *P* < .001) or use free food programs (82.6% vs 74.5%, *P* < .001). We found no changes in dietary intake.

**Conclusion:**

SNAP uptake rates were high after the policy change. Policies that support older adults and adults with disabilities to enroll in or maintain SNAP benefits may improve health outcomes.

**Summary**

**What is already known on this topic?**

Federally funded food assistance programs such as the Supplemental Nutrition Assistance Program (SNAP) reduce food insecurity and improve health in the general population.

**What is added by this report?**

Little is known about how new eligibility for SNAP affects food insecurity and health, especially among older adults and adults with disabilities. We demonstrated that expansion of SNAP eligibility to recipients of Supplemental Security Income (SSI) in California was associated with improvements in food security and general health.

**What are the implications for public health practice?**

Older adults and adults with disabilities are likely to derive substantial benefit from SNAP enrollment. Policies that streamline the receipt and maintenance of benefits may improve the health of older adults and adults with disabilities.

## Introduction

The Supplemental Nutrition Assistance Program (SNAP) is the largest federally funded food assistance program operated by the US Department of Agriculture (1). SNAP improves food access, reduces food insecurity, and decreases poverty among eligible low-income households (2,3).

About 11% of US households were food insecure in 2018, with 4.3% of US households experiencing the most severe form of food insecurity (“very low food security”) (4,5). SNAP benefits decreased rates of food insecurity by about 30% in 2018 (4). From 2016 through 2018, as a result of SNAP benefits, more than 3.5 million people, about 1% of Americans, rose above the federal poverty threshold (4,5).

A robust body of literature demonstrates that SNAP also improves health outcomes (6). These studies showed that SNAP enrollment was associated with decreases in risk of chronic disease, risk of cost-related medication nonadherence among adults with diabetes, hospitalizations and nursing home placements among older adults, and visits to the emergency department for pregnancy-related diagnoses, hypertension, hypoglycemia, and childhood asthma (7–14).

However, SNAP’s capacity to drive these positive outcomes is limited in some populations. Many food-insecure people in the United States, such as people with incomes above the eligibility threshold and undocumented immigrants, are not eligible for SNAP benefits (7). The minimum SNAP benefit level is generally $16 per month in California, but some exceptions allow even lower benefit amounts. In California, beneficiaries of Supplemental Security Income (SSI) — low-income older adults and adults with disabilities — have not been eligible to receive SNAP benefits. Instead, these populations have been able to use a cash benefit provided by SSI to cover food expenses. However, the value of this cash benefit has not increased over time. Thus, many SSI recipients who were otherwise eligible for SNAP benefits because of low household income were excluded in California.

California Assembly Bill 1811 reversed this eligibility policy, allowing California’s SSI recipients to be newly eligible for SNAP (known as CalFresh in California) effective June 1, 2019. Given the positive effect of SNAP in other populations, we sought to determine whether this policy change was associated with changes in food insecurity and health among people receiving SSI in California.

## Methods

We conducted a pre/post study among SSI recipients living in the San Francisco Bay Area. We recruited participants from a network of low-income and supportive housing sites, congregate meal sites, and SNAP outreach events. Participants were also recruited by direct outreach via newsletters, flyers, and referrals. Inclusion criteria were 1) being aged 18 years or older, 2) receiving SSI, 3) not receiving SNAP benefits at baseline, 4) being able to complete the survey in English, and 5) having access to a telephone to complete dietary recalls. Participants who met all criteria but were cognitively impaired (defined as dementia, mental illness, or active substance abuse severe enough to render the person incapable of providing informed consent) were excluded from study participation. Of 236 SSI recipients recruited, we excluded 23 because they received SNAP at baseline (n = 15) or declined to participate (n = 8).

After eligibility was confirmed, we invited potential participants to an in-person orientation to review study details. People who provided informed consent then completed a baseline survey that included questions about demographic characteristics, food insecurity, health status, stress, medication adherence, and knowledge of the new policy. All participants were asked to complete three 24-hour dietary recalls, the gold-standard for assessing dietary intake, over the telephone after the baseline survey. Baseline study participation occurred from May 2019 through August 2019.

Follow-up occurred after the policy change and 4 to 6 months after completion of the baseline survey (September 2019–January 2020). The follow-up survey was administered either by study staff members via telephone or self-administered in person at various outreach sites. We attempted 3 follow-up dietary recalls for each participant.

Participants received a $10 gift card for completion of each survey and, depending on the number completed, $10 to $20 for dietary recalls. The study was approved by the University of California San Francisco Institutional Review Board.

### Measures

**Sociodemographic measures.** Participants were asked to self-report age, sex/gender (male, female, or other), race/ethnicity (American Indian/Alaska Native, Asian, Black/African American, Hispanic/Latino, Native Hawaiian/Pacific Islander, White, or other), highest level of education (<high school, high school graduate/GED, some college/vocational degree, or ≥college graduate), veteran status (yes/no), employment status (yes/no), annual household income (≥$15,000 or <$15,000), and housing status (currently housed or unstably housed). We defined “currently housed” as renting, owning, or living in a single room occupancy unit/motel/hotel, low-income housing, or subsidized housing. We included residents of single room occupancy units/motels/hotels in the “currently housed” category because these types of housing are often used as a permanent housing strategy in San Francisco, where the cost of living is high. We considered participants who identified as homeless or were living in a shelter or “staying for free at someone else’s house” to be unstably housed.

**Outcomes.** Our primary outcome was food security. We scored the 6-item version of the US Department of Agriculture’s US Household Food Security Survey Module: Six-Item Short Form as a dichotomous variable: food secure (0 or 1 item answered affirmatively) or food insecure (2–6 items answered affirmatively) (15). Among participants who were food insecure at baseline, we defined those who became food secure at follow-up as “newly food secure” and those who remained food insecure at follow-up as “persistently food insecure.” To increase the statistical power of our study, we also scored the food security module as a continuous variable (with values ranging from 2.86 to 8.48 and higher values indicating greater food insecurity) by using the US Department of Agriculture’s published weights derived from a Rasch model (15).

Secondary outcomes were stress (measured by the Perceived Stress Scale, which has 10 items scaled 0–40: low, 0–13; moderate, 14–26; high, 27–40) (16), general health status (excellent/very good or good/fair/poor) (17), health-related quality of life reported as number of unhealthy days (4-item CDC Healthy Days Measure, scaled 0–30 unhealthy days of the month) (17), cost-related medication nonadherence (3-item scale for skipping medications to save money, taking less medicine to save money, or delaying filling a prescription to save money: yes, 3, no, 0; not scored, 1 or 2) (18), food trade-offs (4 items assessing trade-offs between food and medical care, utilities, housing, and transportation) (19), use of free community food resources in the past 30 days (including free food program, free groceries, free dining room/soup kitchen, or free home-delivered meals), and average weekly food budget shortfall (1 item) (20). All variables were measured at baseline and at follow-up.

We calculated scores for the Healthy Eating Index–2015 and the alternative Healthy Eating Index–2010 from dietary recalls. The Healthy Eating Index–2015 was calculated as described previously; components were weighted equally across food groups for a maximum score of 100 (21). The alternative Healthy Eating Index–2010, which more strongly predicts chronic disease risk, was also calculated by using a previously described scoring algorithm (22).

In addition, at baseline we asked participants about their familiarity with the upcoming SNAP policy change, familiarity with SNAP, and confidence in enrolling in SNAP (none/somewhat vs moderate/very). At follow-up, we asked participants about their familiarity with the SNAP policy change, their familiarity with SNAP, and their satisfaction with SNAP benefit levels.

### Statistical analysis

We examined changes in variables of interest from baseline to follow-up among all participants and conducted sensitivity analyses among only participants who enrolled in SNAP. We stratified results according to whether participants remained persistently food insecure or became food secure and whether food security scores improved from baseline to follow-up among persistently food-insecure participants.

To accommodate nonnormally distributed data, we analyzed data by using nonparametric statistical tests and SPSS version 24 (IBM Corporation). We used means and ranges to describe continuous variables. Baseline and follow-up data were analyzed by using Mann–Whitney *U* tests for continuous variables and Fisher exact tests for categorical variables. Analyses were conducted to detect differences at baseline and follow-up among all participants, at baseline between newly secure and persistently insecure participants, and at follow-up after adjusting for baseline differences. We conducted the last analysis by using a difference-in-differences strategy that compared newly food-secure and persistently food-insecure participants. We adjusted all multivariable analyses for age, sex/gender, race/ethnicity, and education. A *P* value ≤.05 was considered significant.

## Results

Of the 213 SSI recipients who completed the baseline survey, 157 (73.7%) completed a follow-up survey; the mean time to follow-up was 4.7 months (range, 3.7–7.5 mo). We found no significant differences in any variables (age, sex/gender, race/ethnicity, education, employment, veteran status, food insecurity, health status, and food trade-offs) between participants who did and did not complete a follow-up survey. Overall, 153 of 213 (71.8%) participants completed at least 1 dietary recall at baseline. Participants who completed at least 1 dietary recall completed an average of 2.8 baseline recalls. Almost all (152 of 157) participants at follow-up completed at least 1 recall at follow-up, with an average of 2.7 follow-up recalls per participant. We found no significant differences in variables between participants who did and did not complete at least 1 dietary recall.

Participants at baseline were 56.8% male, 43.7% Black/African American, 11.3% American Indian/Alaska Native, and 8.0% Hispanic/Latino; 11.3% were living in a household with annual household income of $15,000 or more (Table 1). Most participants were food insecure at baseline (83.1%) and currently housed (89.7%); 10.3% were unstably housed.

**Table 1:** Baseline Characteristics of Participants (N = 213) in Study of SNAP Eligibility, Food Security, and Health After a SNAP Policy Change, California, 2019–2020^a^

Characteristic	Value
**Age, y**	
<50	41 (19.2)
50–59	71 (33.3)
60–69	80 (37.6)
70–79	17 (8.0)
Missing data	4 (1.9)
**Sex/gender**	
Male	121 (56.8)
Female	88 (41.3)
Other	3 (1.4)
Missing data	1 (0.5)
**Race/ethnicity^b^**	
American Indian/Alaska Native	13 (6.1)
Asian	5 (2.3)
Black/African American	80 (37.6)
Hispanic/Latino	17 (8.0)
Native Hawaiian/Pacific Islander	1 (0.5)
White	73 (34.3)
Don’t know/unknown/other	41 (19.3)
**Education**	
<High school diploma	54 (25.4)
High school graduate/GED	50 (23.5)
Some college/vocational degree	76 (35.7)
≥College graduate	31 (14.6)
Missing data	2 (0.9)
**Veteran status**	
Veteran	31 (14.6)
Nonveteran	179 (84.0)
Data missing	3 (1.4)
**Employment status**	
Employed	8 (3.8)
Not employed	203 (95.3)
Missing data	2 (0.9)
**Housing status^c^**	
Currently housed	191 (89.7)
Unstably housed	22 (10.3)
**Annual household income, $**	
≥15,000	24 (11.3)
<15,000	187 (87.8)
Missing data	2 (0.9)
**General health status**	
Excellent/very good	57 (26.8)
Good/fair/poor	156 (73.2)
**Healthy Eating Index, mean (median)^d^**	
Healthy Eating Index–2015	44.3 (43.8)
Alternative Healthy Eating Index–2010	45.4 (45.9)
**No. of unhealthy days in past 30 days, mean (median)**	17.1 (20.0)
**Stress score, mean (median)^e^**	
Mean (median)	19.8 (20.0)
Low	28 (13.1)
Medium	145 (68.1)
High	29 (13.6)
Missing data	11 (5.2)
**Food insecurity**	
Food secure	36 (16.9)
Food insecure	177 (83.1)
**Cost-related medication nonadherence**	
Yes	41 (19.2)
No	129 (60.6)
Missing data	43 (20.2)
**Trade-offs^f^**	
Mean (median)	1.3 (0)
Made trade-offs between food and medicine/medical care	76 (39.4)
Made trade-offs between food and utilities	65 (35.9)
Made trade-offs between food and housing	65 (34.6)
Made trade-offs between food and transportation	67 (35.8)
**Use of community food resources in past 30 days**	
Overall	171 (83.0)
Free groceries	110 (64.3)
Free dining room/soup kitchen	92 (53.8)
Home delivered meals	25 (14.6)
**Weekly food budget shortfall (n = 171); mean, median, $**	73.33 (50.00)

Abbreviation: SNAP, Supplemental Nutrition Assistance Program.

a Baseline survey administered to Supplemental Security Income recipients during May–August 2019; policy change in effect beginning June 1, 2019. All values are number (percentage) unless otherwise indicated; percentages may not sum to 100 because of rounding.

b Participants can be both Hispanic and one of the races.

c “Currently housed” defined as renting, owning, living in a single room occupancy unit/motel/hotel, low-income housing, or subsidized housing. “Unstably housed” defined as homeless, living in a shelter, or living in “someone else’s house.”

d Scored from 0 to 100 with higher numbers indicating more nutritious dietary intake; 153 participants answered question; total of 423 dietary recalls.

e Scored from 0 to 40: low, 0–13; moderate, 14–26; high, 27–40.

f Trade-offs defined as answering yes to 1 or 2 times per year, some months, or every month (compared with never); 10.4%–16.6% of data for these variables were missing; percentages based on number who answered question.

The percentage of food-secure participants increased from 16.9% at baseline to 32.5% at follow-up (*P* < .001) (Table 2). The average amount of additional money per participant needed to cover all household food needs for the week decreased from $73.33 at baseline to $47.72 at follow-up (*P* < .001). From baseline to follow-up, the percentage of participants who reported excellent or very good health increased (26.8% vs 27.6%; *P* < .001), and the percentage of participants who reported cost-related medication nonadherence decreased (24.1% vs 17.7%; *P* < .001). A smaller percentage of participants at follow-up also used free community food resources (82.6% vs 74.5%; *P* < .001).

**Table 2:** Change in Outcomes Associated With New SNAP Eligibility Among SSI Recipients in Study of SNAP Eligibility, Food Security, and Health After a SNAP Policy Change, California, 2019–2020^a^

Factor	Baseline (n = 213)	Follow-up^b^ (n = 157)	*P* Value^c^
Food insecurity, n (%)			
Food secure	36 (16.9)	51 (32.5)	<.001
Food insecure	177 (83.1)	106 (67.5)	
Healthy Food Index–2015, mean score^d^	44.3	43.6	.57
Alternative Healthy Food Index–2010, mean score^d^	45.4	44.8	.20
Stress, mean score^e^	19.8	18.5	.32
Mean no. of unhealthy days in past 30 days	17.1	16.5	.96
General health status excellent/very good, n (%)	57 (26.8)	43 (27.6)	<.001
Mean no. of trade-offs^f^	1.3	1.4	.82
Cost-related medication nonadherence, n (%)^g^	41 (24.1)	23 (17.7)	.001
Weekly food budget shortfall, mean, $	73.33	47.72	<.001
Used community food resources in past 30 days, n (%)	171 (83.0)	117 (75.5)	<.001

Abbreviations: SNAP, Supplemental Nutrition Assistance Program; SSI, Supplemental Security Income.

a Baseline survey administered to Supplemental Security Income recipients during May–August 2019; follow-up survey administered September 2019–January 2020. Policy change in effect beginning June 1, 2019.

b Among this group, 72.6% (n = 114) had received SNAP at time of follow-up survey.

c Fisher exact test used for bivariate variables and Mann–Whitney *U* test used for continuous variables.

d Scored from 0 to 100 with higher numbers indicating more nutritious dietary intake.

e Scored from 0 to 40: low, 0–13; moderate, 14–26; high, 27–40.

f Trade-offs defined as answering yes to 1 or 2 times per year, some months, or every month (compared with never).

g Denominator is number of participants who answered question.

At the time of the follow-up survey, 43 (27.4%) participants had not received benefits (including 8.9% who had unsuccessfully attempted to enroll and 18.5% who had not attempted to enroll in SNAP), 50 (31.8%) had received SNAP benefits for 0 to 2 months, 59 (37.6%) had received SNAP benefits for more than 3 months, and 5 (3.2%) had received SNAP benefits for an unknown period of time. The mean self-reported SNAP benefit among those successfully enrolled was $73.50 per month (range, $6.00–$345.00). Among the 114 participants who answered a question about satisfaction with SNAP benefit levels, 24 participants (21.1%) reported the amount of SNAP benefit they received each month was “about right” and others reported receiving benefits at a level that was “a little too low” (n = 38; 33.3%) or “way too low” (n = 49; 43.0%); no participants reported benefit levels that were “a little high” or “way too high.” Among participants who received SNAP, greater monthly SNAP benefits correlated with a smaller weekly food budget shortfall (*P* < .001) (Figure).

**Figure:**
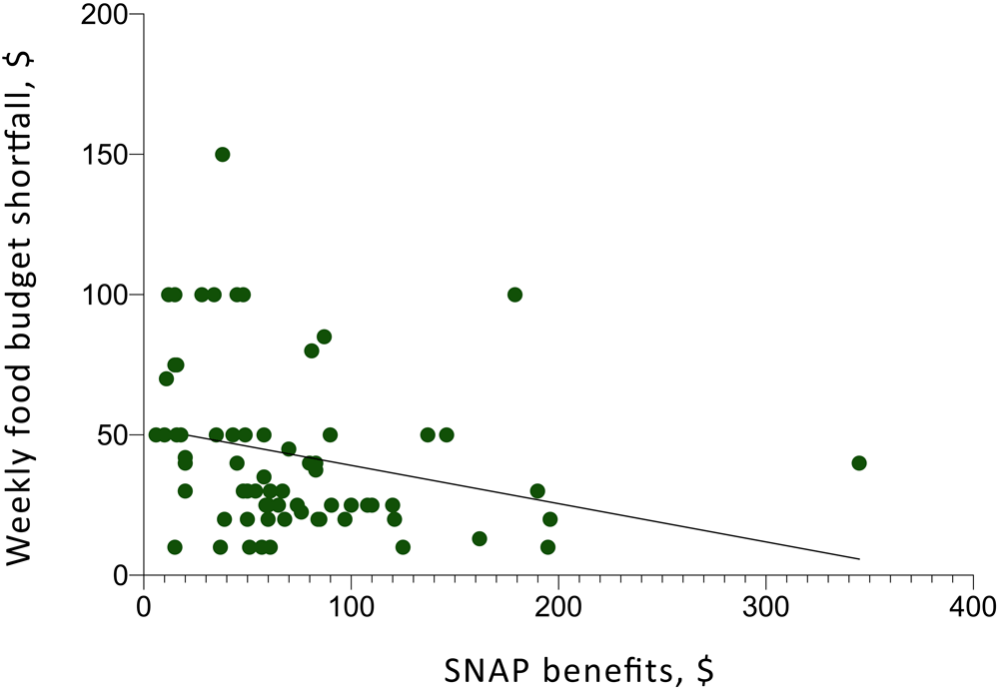
Correlation between SNAP benefits and weekly budget shortfall at follow-up. All units are US dollars. A line of best fit has a negative slope and an *r* ^2^ of 0.066. Abbreviation: SNAP, Supplemental Nutrition Assistance Program.

Of the 128 participants who were food insecure at baseline, 28 (21.9%) participants became newly food secure and 98 (76.6%) participants remained persistently food insecure. We found no significant differences in demographic characteristics or SNAP benefit factors (amount of money received in SNAP benefits or length of time receiving SNAP benefits) between participants who became newly food secure and participants who remained persistently food insecure. However, participants who became newly food secure had reported less severe food insecurity at baseline than participants who were persistently food insecure (6.7 vs 7.2; *P* = .003). At follow up, compared with persistently food-insecure participants, newly food-secure participants reported having less stress (*P* = .02) in the difference-in-differences analysis (Table 3). Newly food-secure participants also reported more familiarity with changes to SNAP policy than persistently food-insecure participants (78.6% vs 52.6%; *P* = .02) but were not more likely to receive SNAP (75.0% vs 72.4%; *P* > .99). Receipt of SNAP did not predict becoming newly food secure at follow-up either in bivariate analysis (*P* = .83) or after adjusting for sex/gender, education, age, and race/ethnicity (*P* = .11).

**Table 3:** Baseline and Follow-up Differences Between Participants Who Were Newly Food Secure and Participants Who Were Persistently Food Insecure in Study of SNAP Eligibility, Food Security, and Health After a SNAP Policy Change, California, 2019–2020^a^

Item	Persistently Insecure (n = 100)	Newly Secure (n = 28)	*P* Value^b^
**Baseline factor**			
Food insecurity, mean^c^	7.2	6.7	.003
Healthy Food Index–2015, mean score^d^	45.6	43.2	.25
Alternative Healthy Food Index–2010, mean score^d^	46.9	44.3	.25
Stress, mean score^e^	20.1	16.7	.08
Mean no. of unhealthy days	16.9	11.9	.06
General health status excellent/very good, n (%)^b^	74 (75.5)	16 (57.1)	.10
Mean no. of trade-offs^f^	1.9	1.5	.08
Cost-related medication nonadherence, n (%)	26 (34.7)	4 (18.2)	.19
Weekly food budget shortfall, mean, $	80.00	54.70	.25
Used community food resources in past 30 days	78 (83.0)	24 (85.7)	>.99
**Difference in difference from baseline to follow-up**			
Food insecurity, mean^c^	−0.1	−5.3	<.001
Healthy Food Index–2015, mean score^d^	−0.3	−2.6	.49
Alternative Healthy Food Index–2010, mean score^d^	−2.1	−2.5	.54
Stress, mean score^e^	0.7	−9.0	.02
Mean no. of unhealthy days	2.1	−10.3	.52
General health status excellent/very good, n (%)^b^	8 (8.2)	1 (3.6)	.68
Mean no. of trade-offs^f^	−0.3	−1.3	.53
Cost-related medication nonadherence, n (%)	7 (11.5)	1 (4.5)	.68
Weekly food budget shortfall, mean, $	−34.76	−29.67	.87
Used community food resources in past 30 days, n (%)	7 (7.4)	1 (3.6)	.68

Abbreviations: SNAP, Supplemental Nutrition Assistance Program.

a Baseline survey administered to Supplemental Security Income recipients during May–August 2019; follow-up survey administered September 2019–January 2020. Policy change in effect beginning June 1, 2019.

b Fisher exact test used for bivariate variables and Mann–Whitney *U* test used for continuous variables.

c The US Department of Agriculture’s US Household Food Security Survey Module: Six-Item Short Form was scored as a continuous variable (minimum value, 2.86; maximum, 8.48) by using the US Department of Agriculture’s published weights derived from a Rasch model (15); the higher the score, the greater the food insecurity.

d Scored from 0 to 100 with higher numbers indicating more nutritious dietary intake.

e Scored from 0 to 40: low, 0–13; moderate, 14–26; high, 27–40.

f Trade-offs defined as answering yes to 1 or 2 times per year, some months, or every month (compared with never).

Among the 98 participants who remained persistently food insecure at follow-up, regardless of SNAP status, food-insecurity scores improved (paired sample mean range, from 7.4 to 7.2; *P* < .001). Receipt of SNAP did not predict improvement in food-security scores at follow-up either in bivariate analysis (*P* = .83) or after adjusting for sex/gender, education, age, and race/ethnicity (*P* = .55).

With the exception of employment, baseline demographic characteristics were not significantly different between participants who received SNAP benefits and participants who did not receive SNAP benefits. Participants who received SNAP benefits were less likely to be employed at baseline than participants who did not receive SNAP benefits (2 participants [1.8%] vs 4 participants [9.3%], *P* = .048). All outcomes were similar between participants who received SNAP benefits and participants who did not receive SNAP benefits (Table 4).

**Table 4:** Change in Outcomes Associated With Receipt of SNAP Benefits at Follow-Up Among SSI Recipients in Study of SNAP Eligibility, Food Security, and Health After a SNAP Policy Change, California, 2019–2020^a^

Factor	Did Not Receive SNAP Benefits (n = 43)	Received SNAP Benefits^b^ (n = 114)	*P* Value^c^
Food insecurity, n (%)			
Food secure	15 (34.9)	36 (31.6)	.71
Food insecure	28 (65.1)	78 (68.4)	
Healthy Food Index–2015, mean score^d^	45.8	45.0	.57
Alternative Healthy Food Index–2010, mean score^d^	44.3	46.7	.20
Stress, mean score^e^	20.5	19.1	.65
Mean no. of unhealthy days	17.4	16.0	.69
General health status excellent/very good, n (%)^d^	10 (23.3)	33 (29.2)^f^	.55
Mean no. of trade-offs^g^	1.7	1.5	.45
Cost-related medication nonadherence, n (%)	6 (17.6)	17 (17.7)^f^	>.99
Weekly food budget shortfall, mean, $	73.68	41.85	.48
Used community food resources in past 30 days, n (%)	29 (70.7)	88 (77.2)	.41

Abbreviations: SNAP, Supplemental Nutrition Assistance Program; SSI, Supplemental Security Income.

a Baseline survey administered to Supplemental Security Income recipients during May–August 2019; follow-up survey administered September 2019–January 2020. Policy change in effect beginning June 1, 2019.

b Of this group, 72.6% (n = 114) had received SNAP at time of follow-up survey.

c Fisher exact test for bivariate variables and Mann–Whitney *U* Test for continuous variables.

d Scored from 0 to 100 with higher numbers indicating more nutritious dietary intake.

e Scored from 0 to 40: low, 0–13; moderate, 14–26; high, 27–40.

f Not all participants answered all questions; percentages based on number who answered question.

g Trade-offs defined as answering yes to 1 or 2 times per year, some months, or every month (compared with never).

## Discussion

Most SSI recipients we sampled successfully enrolled in SNAP in response to the June 2019 policy change that expanded eligibility to SSI recipients. Overall, compared with participants at baseline, participants at follow-up were more food secure and had better general health status, lower weekly food budget shortfall, less use of free food programs, and less cost-related medication nonadherence.

We did not observe a difference in outcomes between participants who received SNAP benefits and participants who did not receive SNAP benefits, which may be due to the small number of participants who did not receive SNAP benefits at follow-up. (High levels of program uptake is likely the result of robust outreach by local SNAP offices and suggests that SNAP is desirable in this population.) However, the changes in outcomes observed from baseline to follow-up may be related at least in part to SNAP enrollment, as most participants received SNAP at follow-up. This conclusion is supported by multiple other observations. First, participants who received higher SNAP benefit levels reported lower weekly food budget shortfalls. Second, a smaller percentage of participants at follow-up used free food program resources, suggesting that improvements observed were not related to use of programs other than SNAP.

The association between SNAP and improved general health and reduced cost-related medication nonadherence is consistent with previous research that examined the effect of interventions designed to improve food security (7–14). Our findings therefore extend the current literature on SNAP and its effect on health outcomes by suggesting that SSI recipients can also derive substantial health benefits from SNAP eligibility.

SNAP tends to affect the severity of poverty more than prevalence of poverty (3); its effects are greatest among those with the deepest poverty levels, but benefit levels in those households may be inadequate to allow a crossing of the poverty threshold. A similar phenomenon may have occurred in our study — many participants at follow-up improved their food insecurity score, but the improvements were insufficient to cross the threshold into food security. This hypothesis is supported by several lines of evidence. First, even among persistently food-insecure participants, continuous scores of food insecurity improved. Second, participants who were newly food secure at follow-up were less food insecure at baseline than those who remained food insecure at follow-up. Finally, the weekly food budget shortfall improved even among those participants who did not become food secure.

New food security was associated with lower levels of stress but not with improvements in other health outcomes, including general health status, number of unhealthy days, reliance on free food programs, or trade-offs between food and other basic necessities. Therefore, although minimal improvement in food insecurity, possibly as a result of SNAP enrollment, has numerous benefits, our findings suggest that additional improvements in health, lifestyle, and dietary intake may require higher benefit levels or additional interventions.

We did not observe improvements in dietary intake associated with the policy change. Low-income older adults and adults with disabilities often have additional barriers to healthy dietary intake in addition to food insecurity, including limitations in transportation, equipment to store and prepare food, and physical capacity to cook. SNAP enrollment may therefore address some, but not all, of the barriers preventing healthy dietary intake (23). Thus, SNAP benefits may be necessary but not sufficient for improving dietary intake in this population.

Our study has several limitations. First, variables other than changes in SNAP policy likely exist to explain the improvements in food security we observed. Although we found no significant difference in the self-reported number of unhealthy days between persistently food-insecure and newly food-secure participants at baseline or in the difference-in-differences analysis, participants who became newly food secure at follow-up may have had advantages, such as more resources or better health, that persistently food-insecure participants did not have and we did not measure.

Second, we recruited participants from a single urban setting, and most participants enrolled in SNAP soon after the policy change. Thus, our findings may not generalize to other populations. Third, biases are associated with the recruitment of participants primarily from housing sites and only those who are proficient in English. Some participants were also recruited from SNAP outreach events, skewing our sample to people more familiar with the SNAP policy change and more likely to participate in SNAP after the policy change went into effect. Fourth, almost half of participants had received SNAP benefits for less than 2 months at the time of their follow-up survey, which may have limited the time available for the intervention to significantly affect health and other outcomes.

Despite these limitations, our study contributes important findings to the SNAP literature. To our knowledge, ours is the first study to explore changes in food security among SSI recipients before and after new eligibility for SNAP. Our survey reached a population of adults at high risk of health decline, hospitalizations, and institutionalization. Additionally, our study provides valuable insight into factors associated with improvements in food security among SSI participants enrolled in SNAP and differential ways in which the opportunity to enroll in SNAP may affect people for whom benefit levels are adequate to achieve food security.

Disability is one of the strongest risk factors for food insecurity, and households with adults with disabilities have more severe food insecurity than households without adults with disabilities (24,25). We found that expansion of SNAP benefits to SSI recipients was associated with improved food security. Thus, interventions such as expansion of SNAP eligibility may be particularly important for reducing inequities in health outcomes in this population.

Future research should focus on exploring other variables that may improve food security among older adults and adults with disabilities. Additionally, future studies may benefit from longer duration between enrollment and follow-up to better examine the long-term potential benefits, including the health benefits, of SNAP expansion to newly eligible populations. Given that we did not observe changes in dietary intake in our study, future research could also explore the potential of SNAP programs that feature incentives for consuming fruits and vegetables. The change in SNAP eligibility investigated in our study is the kind of natural experiment that offers an important opportunity to examine the effect of SNAP on food security, health behaviors, and health outcomes.
